# Protein-Protein Interactions Prediction Based on Graph Energy and Protein Sequence Information

**DOI:** 10.3390/molecules25081841

**Published:** 2020-04-16

**Authors:** Da Xu, Hanxiao Xu, Yusen Zhang, Wei Chen, Rui Gao

**Affiliations:** 1 School of Mathematics and Statistics, Shandong University, Weihai 264209, China; daxusdu@163.com (D.X.); hanxiaoxusdu@163.com (H.X.); chenwei@sdu.edu.cn (W.C.); 2 School of Control Science and Engineering, Shandong University, Jinan 250061, China; gaorui@sdu.edu.cn

**Keywords:** protein-protein interaction, graph energy, physicochemical properties, contact information, WSRC classifier

## Abstract

Identification of protein-protein interactions (PPIs) plays an essential role in the understanding of protein functions and cellular biological activities. However, the traditional experiment-based methods are time-consuming and laborious. Therefore, developing new reliable computational approaches has great practical significance for the identification of PPIs. In this paper, a novel prediction method is proposed for predicting PPIs using graph energy, named PPI-GE. Particularly, in the process of feature extraction, we designed two new feature extraction methods, the physicochemical graph energy based on the ionization equilibrium constant and isoelectric point and the contact graph energy based on the contact information of amino acids. The dipeptide composition method was used for order information of amino acids. After multi-information fusion, principal component analysis (PCA) was implemented for eliminating noise and a robust weighted sparse representation-based classification (WSRC) classifier was applied for sample classification. The prediction accuracies based on the five-fold cross-validation of the human, Helicobacter pylori (*H. pylori*), and yeast data sets were 99.49%, 97.15%, and 99.56%, respectively. In addition, in five independent data sets and two significant PPI networks, the comparative experimental results also demonstrate that PPI-GE obtained better performance than the compared methods.

## 1. Introduction

Protein-protein interaction (PPI) plays a distinctly important role in understanding cellular biological activities [1]. Its research contributes to understanding the protein function, mechanism of biological activity, disease diagnosis and prevention, and new drug development [2,3,4]. The research methods of PPI can be divided into two types: computational and experimental methods. Over the past few decades, many innovative experimental technologies have been designed to attempt to validate PPIs, such as glutathione *S*-transferase [5], protein chip [6], yeast two-hybrid [7], tandem affinity purification (TAP) tag [8], and other high-throughput technologies. Some direct interactions data of different species have been discovered and validated [9]. However, the traditional experiment-based methods are not only costly and time-consuming, but also have high rates of false-positive predictions and weak generalization ability. Therefore, developing new reliable computational approaches has great practical significance for PPI identification at low cost and high efficiency [10].

In recent years, some computational approaches based on various types of information about proteins have been suggested to predict PPIs, such as genomic information, structure information, evolutionary knowledge, protein domains, and phylogenetic profiles [11,12,13,14]. However, the above prior information that can be used to predict the PPIs is scarce compared with the rapid growth of amino acid sequences [15]. The above methods cannot be applied without prior knowledge of the proteins. In contrast, it is more significant to only use the protein amino acid sequences for predicting PPIs.

Extensive experiments show that using the protein sequence information alone is enough for identifying PPIs [16]. Many sequence-based computational methods have been explored to address the problems, such as support vector machine (SVM) with multi-scale discontinuous and continuous [16], rotation forest algorithm with position-specific scoring matrix (PSSM) [17], SVM with auto covariance (AC) [18], average blocks (AB) using relevance vector machine (RVM) [19], discrete cosine transformation using weighted sparse representation [20], and so on. In 2018, Göktepe et al. [21] presented a feature representation method named weighted skip-sequential conjoint triads using principal component analysis (PCA) and SVM to capture the information of protein sequences. In 2019, Chen et al. [1] designed an end-to-end framework which incorporated a deep residual recurrent convolutional neural network for capturing the information of protein sequences. In the same year, Zhang et al. [22] presented a neural network-based model which separately used different descriptors (auto covariance descriptor, local descriptors, and multi-scale continuous and discontinuous local descriptor) to explore and represent the patterns of interactions between amino acids. Although the researches of these approaches have achieved good progress and application prospects, new methods are needed to further improve the performances of PPI predictions.

The concept of the energy of graph G is due to Gutman [23] and is meaningful for the analysis of graph theory [24,25,26]. Nowadays, the energy of the graph has been used in chemistry, bioinformatics, and related fields [27,28]. In the literature, increasing studies have shown that the physicochemical properties of amino acids can improve the prediction performances of PPIs [16,29]. The contact information among amino acids is also significant for prediction of PPIs [30]. The multi-information fusion of different feature extraction methods can fuse different feature information of interacting protein sequences, and it is an effective technique in improving the prediction performance of PPIs [22].

In this paper, we present a computational model to predict PPIs using only protein sequences and graph energy. Inspired by the graph energy theory, we design two feature extraction methods for PPIs—physicochemical graph energy (PGE) and contact graph energy (CGE)—to capture the feature information of interactions. Physicochemical graph energy is graph energy based on physical and chemical properties, while contact graph energy is graph energy based on amino acid contact information. The dipeptide composition method was also used to extract and supplement effective information. PCA was implemented to effectively reduce the influence of noise after integrating three feature extraction methods. The weighted sparse representation-based classification (WSRC) was used as the classifier of the proposed method after different classifiers were compared. The PPI-GE has been tested on human, *H. pylori*, and yeast data sets, and these three data sets achieved prediction accuracies of 99.49%, 97.15%, and 99.56%, respectively. In addition, we verified the validity of the proposed method on five independent data sets and two significant PPI networks. The comparative experimental results indicate that our feature extraction methods have a significant effect on PPI prediction and our method is superior to other state-of-the-art prediction methods.

## 2. Results and Discussion

### 2.1. Evaluation Metrics

In this study, to ensure the reliability of experimental results and avoid over fitting of data, we implemented five-fold cross-validation to evaluate the effectiveness of PPI-GE and other computational models. Specifically, the experimental data set was split into five parts; each of the five parts is regarded as an independent testing data set and the other four parts were selected as training data sets. Several widely-used evaluation metrics were used, including accuracy (ACC), sensitivity (SEN), precision (Pre), and Matthews correlation coefficient (MCC), expressed as follows:

(1) $$ACC=\frac{TP+TN}{TP+TN+FP+FN}$$

(2) $$SEN=\frac{TP}{TP+FN}$$

(3) $$Pre=\frac{TP}{TP+FP}$$

(4) $$MCC=\frac{TP\times TN-FP\times FN}{\sqrt{\left(TN+FP\right)\times \left(TP+FP\right)\times \left(TP+FN\right)\times \left(TN+FN\right)}}$$

where *TP*, *TN*, *FP*, and *FN* denote the number of true positives, true negatives, false positives, and false negatives, respectively. In the protein–protein interaction data sets, the unknown protein-protein interactions are considered negative samples, while the known interactions are called positive samples. The average performance of all evaluation metrics is obtained during the experiment. As the value of metric is larger, the performance of the method will be better. Moreover, the area under the receiver-operating characteristics curve (AUC) was calculated to further evaluate the performance of the method. The AUC value of 1 indicates perfect prediction and AUC value of 0.5 means random prediction.

### 2.2. The Performance Comparisons of Different Classifiers

It is well known that the same method using different classifiers may achieve different prediction results. To further evaluate the proposed method, the K-nearest neighbors (KNN), support vector machine (SVM), and WSRC classifiers were adopted to predict PPIs using the same feature extraction methods. To ensure the universality of different data set, we implemented five-fold cross-validation 10 times and obtained the average value of three benchmark data sets for the same evaluation metric using every classifier, respectively.

**Figure 1:**
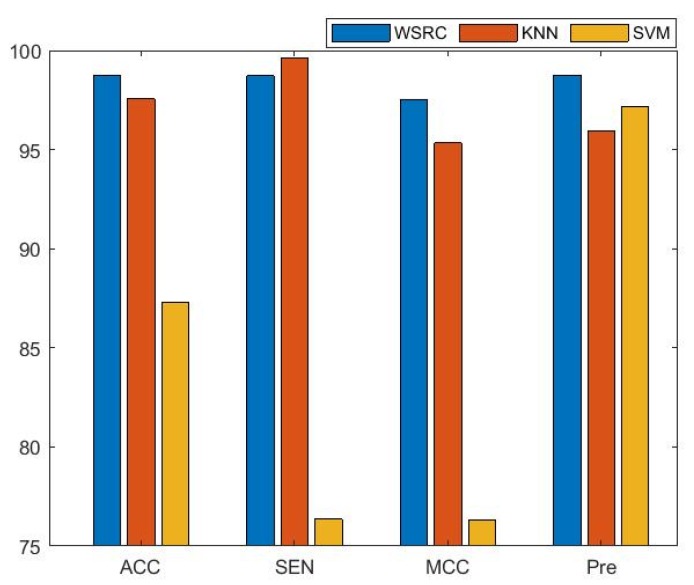
The performance comparisons of different classifiers. WSRC: weighted sparse representation-based classification; KNN: K-nearest neighbors; SVM: support vector machine.

The average results of three benchmark data sets with different classifiers are presented in Figure 1. From the figure, the results of the comparison prove that the performance of the WSRC classifier has better stability and higher accuracy than the SVM and KNN classifiers for predicting PPIs. In this work, we used the WSRC classifier as the classifier of our model.

### 2.3. Prediction Performances of the Proposed Method

For verifying the efficacy and stability and reducing deviations of PPI-GE based on the WSRC classifier, five-fold cross-validation was performed in the experiment. The cross-validation results of three benchmark data sets are shown in Table 1, Table 2 and Table 3.

When performing on the human data set, ACC, SEN, MCC, Pre, and AUC achieved the average performance of 99.49%, 99.21%, 98.97%, 99.72%, and 99.99%, respectively (see Table 1). Similarly, the average results of ACC, SEN, MCC, Pre, and AUC on the *H. pylori* data set were 97.15%, 98.23%, 94.35%, 96.17%, and 99.19%, respectively (see Table 2). At the same time, we also gained better average results of these metrics at 99.56%, 99.14%, 99.13%, 99.98%, and 100% on the yeast data set, respectively (see Table 3). The experimental results show that PPI-GE is robust and promising for predicting PPIs. Our method achieved better prediction results which may be attributed to the choice of the classifiers and novel feature extraction methods.

### 2.4. Comparison with Other Methods

**Figure 2:**
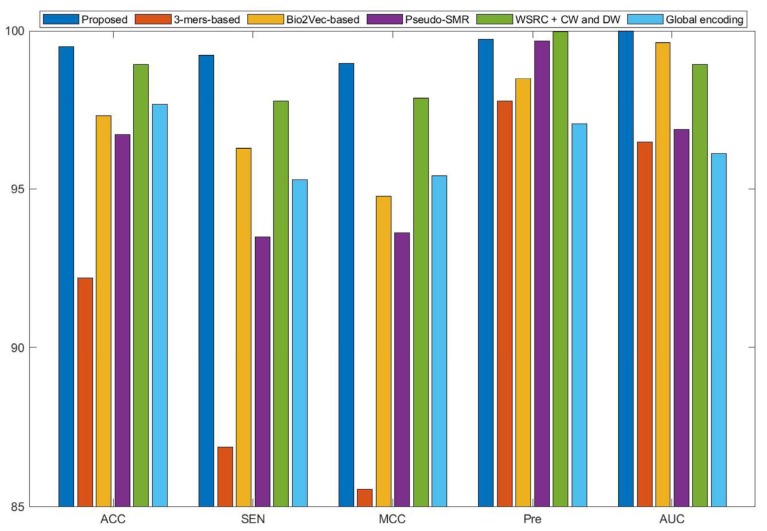
Comparison results of different methods on the human data set.

**Figure 3:**
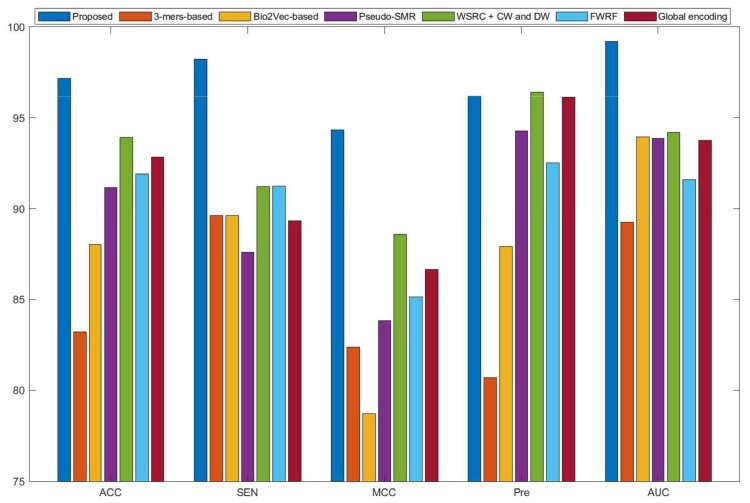
Comparison results of different methods on the *H. pylori* data set.

**Figure 4:**
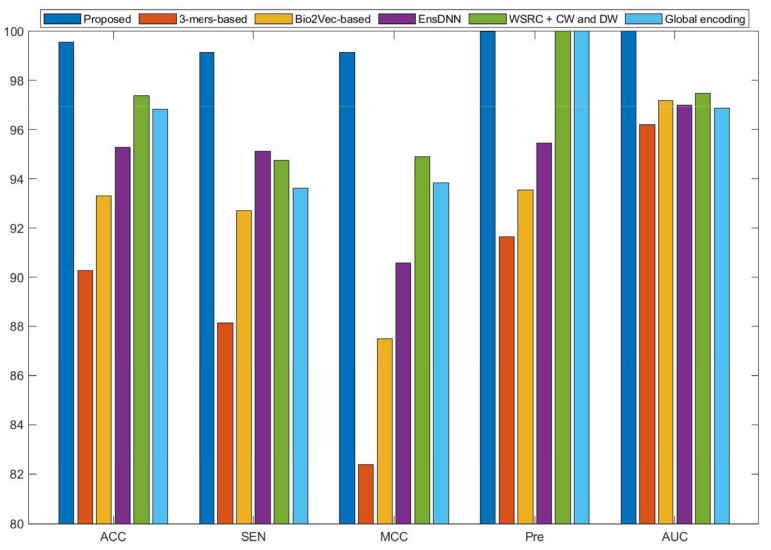
Comparison results of different methods on the yeast data set.

Currently, many kinds of computational models based on protein sequences have been presented for predicting PPIs. In this section, to further objectively validate the prediction performance of the proposed method, seven state-of-the-art methods, including Ensemble Deep Neural Networks (EnsDNN) [22], 3-mers-based [31], Bio2vec-based [31], pseudo Substitution Matrix Representation (pseudo-SMR) [32], WSRC with continuous wavelet and discrete wavelet transform (WSRC+CW and DW) [33], feature weighted rotation forest algorithm (FWRF) [17], and Global encoding [34] were compared on the human, *H. pylori*, and yeast data sets. The comparison results of three benchmark data sets based on five-fold cross-validation of different models are plotted in Figure 2, Figure 3 and Figure 4, respectively.

Some previous algorithms did not use all three benchmark datasets in their papers, therefore we first compared the proposed method with the other five methods on the human data set. Figure 2 shows that the proposed method obtained higher average accuracy (99.49%) out of these methods. Meanwhile, the results of SEN, MCC, and AUC are superior to others. On the *H. pylori* data set, our method and six other methods were used for the comparison. From Figure 3, it can be noted that our method is significantly better than that of others. On the yeast data set, the results of comparison among six different methods are shown in Figure 4. We obtained similar results. The comparison results show that our method obtained satisfactory performance relative to current existing approaches. This further demonstrates that the proposed method based on the novel feature extraction methods is robust and effective.

### 2.5. Performance on PPI Networks

Since the development of the disease may involve proteins and pathways in multiple biological processes, PPI networks may help to understand the deregulated molecular mechanisms of disease development and progression and the functional organization of proteins. The general PPI networks are crossover networks from a biological perspective [35]. It is necessary to evaluate the performance of the proposed method by predicting the PPI network. In this section, the Wnt signaling pathway network is a significant crossover network, which was used for evaluating.

**Figure 5:**
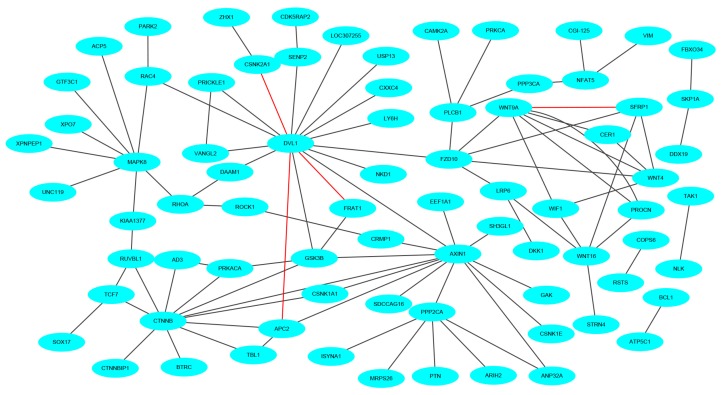
The prediction results of crossover network (Wnt-related network).

**Figure 6:**
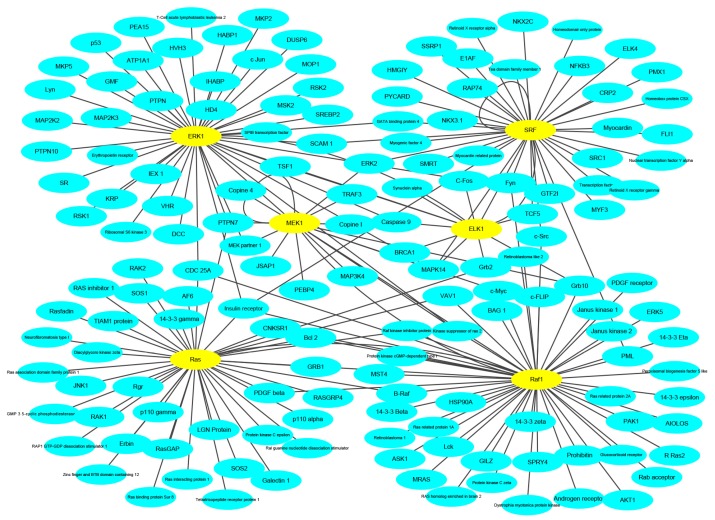
The prediction results of multi-core network.

To keep the same experimental conditions during the comparison, the yeast data set was regarded as the training data set and the Wnt-related network was regarded as the testing data set. Since they are different species, in the encoding, the dimension of fused feature vector E was reduced to 20 for eliminating the influence of more noise. The network and prediction results are shown in Figure 5. The red line is false prediction. It can be seen from the figure that our method can predict 92 of the 96 PPIs. We also compared some previous methods with the proposed method, and the comparisons are listed in Table 4. From the table, it can be noted that the proposed method is significantly better than others. In addition to this, we also tested our method on the multi-core network (Ras-Raf-Mek-Erk-Elk-Srf pathway) for predicting. The network is shown in Figure 6. The core protein is colored yellow. Our method correctly predicts all PPIs. The results suggest that PPI-GE can be applied to predict PPIs encoded in the network and obtain better prediction results.

### 2.6. Performance on Independent Data Sets

Finally, to further validate the efficacy and stability of our method, we also tested the proposed method and compared it with several state-of-the-art methods on five independent data sets (*H. pylori*, *H. sapien*, *C. elegans*, *M. musculus*, and *D. mela* data sets). In the encoding, the yeast data set was regarded as the training data set and the independent data set was regarded as the testing data set, and the same feature extraction methods were used during the experiment. The comparison results between different methods are summarized in Table 5. The accuracies of five independent data sets were 93.80%, 99.93%, 86.24%, 94.57%, and 99.87%, respectively. The proposed method has better performance for PPI prediction on four data sets (*H. pylori*, *H. sapien*, *M. musculus*, and *D. mela*). However, our accuracy on the *C. elegans* data set is lower than Du’s work and Ding’s work. Overall, it indicates that our method can perform across species for PPI predictions.

## 3. Materials and Methods

**Figure 7:**
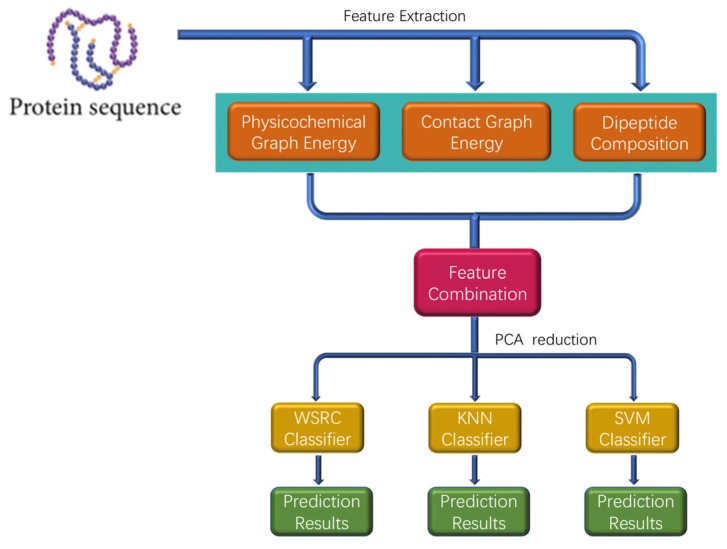
The flowchart of the proposed method for predicting protein-protein interactions (PPIs).

In this section, a novel method called PPI-GE is described, which depends mainly on three steps. The flowchart of PPI-GE is shown in Figure 7. First, the method only uses the amino acid sequences through physicochemical graph energy, contact graph energy, and dipeptide composition for feature extraction and multi-information fusion. Then, the PCA method was implemented for the descending dimension and eliminating noise. Finally, the WSRC classifier was applied for sample classification and predicting PPIs after different classifiers were compared.

### 3.1. Datasets

In this work, three high-quality benchmark data sets were used to ensure generality and evaluate the performance of the proposed method. The first data set is the human data set constructed by Huang et al. [20]. They collected 3899 experimentally verified PPIs as a positive sub-dataset and obtained 4262 non-PPI pairs from different subcellular compartments as a negative sub-dataset. The *H. pylori* data set is used as the second data set constructed by Martin et al. [38], and the third data set is the yeast data set collected by Guo et al. [18]. The summary of three benchmark data sets can be seen in Table 6.

In addition, we tested on two significant PPI networks to objectively validate the performance of the proposed method. The first network is the crossover network (Wnt-related network) [39] which contains 96 PPIs. It is a significant signaling pathway and plays a distinctly important role in the understanding of tumor formation, processes of cytoskeletal organization, patterning, and organogenesis. The second network is the multi-core network (Ras-Raf-Mek-Erk-Elk-Srf pathway) [40] which includes 189 PPIs. It is an important consensus network and implicates a variety of transcriptional regulations and cellular processes. To further verify the efficacy and stability of our method, we also tested it on five independent data sets, including *H. pylori*, *H. sapien*, *C. elegans*, *M. musculus*, and *D. mela* data sets [30,34].

### 3.2. Feature Extraction

In this work, we designed two feature extraction methods to capture the feature information of sequences: the graph energy based on the physical and chemical properties named physicochemical graph energy (PGE) and the graph energy based on the amino acid contact information named contact graph energy (CGE). The concept of the energy of a graph is due to Gutman [23]. If graph G is a simple graph, the energy of the graph is defined as the sum of the absolute eigenvalues of the adjacency matrix of the graph G. If E(G) represents the energy of a graph, we get

(5) $$E\left(G\right)=\overset{n}{\underset{i=1}{\sum }}|\lambda_{i}|$$

where $\lambda_{i}$ is the ith eigenvalue of the adjacency matrix [25,26].

#### 3.2.1. Physicochemical Graph Energy

Amino acids are the basic units of protein sequences and have different physicochemical properties which have a great significance for the prediction of protein functions and structures [41,42]. Conventionally, the location information of the amino acid sequence is important in the prediction of PPIs, because amino acids make up protein sequences and have specific positions which are closely related to the local interaction information of amino acid neighborhoods.

**Figure 8:**
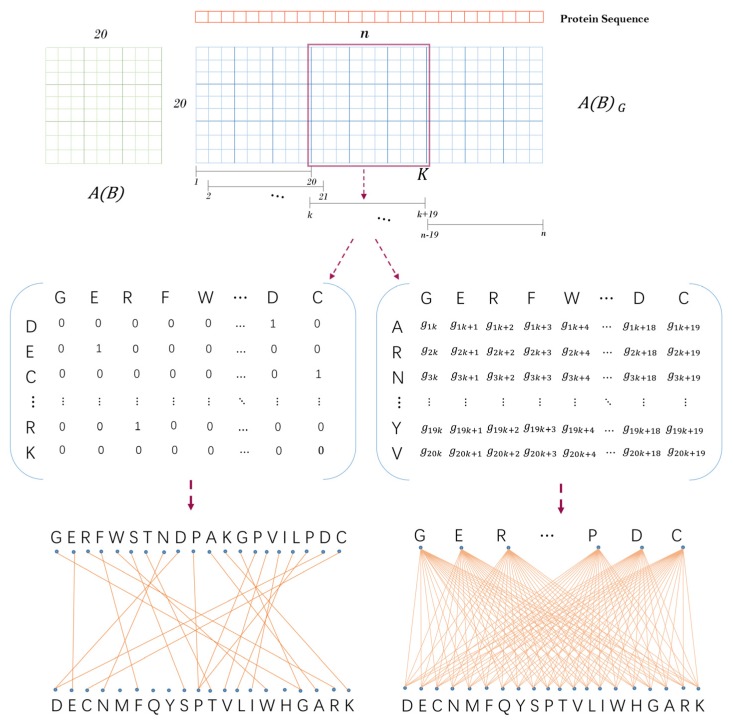
The schematic diagram of protein sequence feature extraction.

Inspired by previous work [27], we obtained a descending order (D→E→C→N→M→F→Q→Y→S→P→T→V→L→I→W→H→G→A→R→K) based on the isoelectric point and ionization equilibrium constant typical physicochemical properties of amino acids. Then, a unit substitution matrix $A\in R^{20\times 20}($as shown in Figure 8) was constructed by using this ordering to describe the location information of amino acids. A protein sequence with the length of n can be transformed into a (0,1)-adjacency matrix $A_{G}={\left(g_{i,j}\right)}_{20\times n}$ based on the unit substitution matrix A. It is defined as follows: when the jth amino acid type of protein sequence is the same as the tth amino acid type of above amino acid order, let $A_{G}\left(i,j\right)=A\left(i,t\right)$, where i,t=1,2,⋯,20; j=1,2,⋯,n.

Next, we constructed a sliding window with the length of 20 to transform the matrix $A_{G}$ into n−19 matrixes of 20×20 dimensions. Let $A_{G}^{k}\in R^{20\times 20}$ be a sliding window which starts from the first amino acid at the left end of the protein sequence and moves the position of one amino acid at a time. Suppose $A_{G}^{k}$ is the sparse sub-matrix obtained by sliding the window to the kth amino acid of the sequence, then $A_{G}^{k}\left(i,j\right)=A_{G}\left(i,k+j-1\right)$, where i=1,2,⋯,20;j=1,2,⋯,20;k=1,2,⋯,n−19. As shown in Figure 8, $A_{G}^{k}$ can correspond to a sparse bipartite graph $G_{Ak}$ one by one, and each amino acid corresponds to a point in the bipartite graph. Finally, the physicochemical graph energy $E\left(G_{Ak}\right)$ of each bipartite graph $G_{Ak}$ was calculated as Equation (5). In this way, we turned a protein sequence into a numerical vector $E_{1}^{*}=\{E\left(G_{A1}\right),E\left(G_{A2}\right),\cdots ,E\left(G_{\mathrm{An}-19}\right)$}.

Machine learning methods need to input feature vectors with the same lengths, but different proteins may have different sequence lengths. In the literature, the top 30-dimensional feature vector of a protein sequence can contain some information for the similarity analysis of proteins [28]. Here, we set 200 windows to obtain enough information of proteins for PPIs. The algorithm implements zero-padding if the length of the protein is less than 219 residues. Thus, a protein sequence with the length of n can be characterized by a 200-dimensional numerical vector $E_{1}^{*}=\{E\left(G_{A1}\right),E\left(G_{A2}\right),\cdots ,E\left(G_{A200}\right)$}.

#### 3.2.2. Contact Graph Energy

The contact information among different types of amino acids is significant for predicting PPIs as described by Ding et al. [30]. They considered 20 kinds of amino acid, 8 types of secondary structures, disability contact solvents, and 6323 complexes [43,44]. Then, the average number of pairwise contacts observed at the interface was calculated from unbound protein to binding structure. In this section, the second alternative matrix $B\in R^{20\times 20}$ (as shown in Figure 8) is the contact matrix of amino acids which is based on the effective contact energy among different types of amino acids. It is noteworthy that we used the same amino acid contact matrix as used in Ding’s work.

Next, as the physicochemical graph energy is based on the substitution matrix B, the protein sequence was transformed into an adjacency matrix $B_{G}\in R^{20\times n}$. We set a sliding window to slide on the protein sequence and obtain sub-matrix $B_{G}^{k}\in R^{20\times 20}$, where k=1,2,⋯,n−19. Each matrix $B_{G}^{k}$ corresponds to a complete bipartite graph one by one. Finally, the contact graph energy $E\left(G_{Bk}\right)$ of each bipartite $G_{Bk}$ was calculated as Equation (5).

Therefore, according to the proposed contact graph energy, we can characterize and transform a protein sequence into a numerical vector $E_{2}^{*}=\{E\left(G_{B1}\right),E\left(G_{B2}\right),\cdots ,E\left(G_{B200}\right)$}.

#### 3.2.3. *N*-peptide Composition Representation

It is generally known that amino acids are the basic units that make up protein sequences. A protein sequence can be simply expressed as follows:

$$P=A_{1}A_{2}\cdots A_{i}\cdots A_{L-1}A_{L}$$

where $A_{i}$ represents the ith amino acid and each belongs to one of the 20 native amino acid types; L denotes the number of amino acids in the sequence.

The pseudo amino acid composition is widely used to extract sequence information of proteins. The simplest one is called n-peptide composition. In this way, it can preserve the protein sequence order information. When n=2, this method degenerates the amino acid composition into a dipeptide composition [21,45,46]. Dipeptide composition treats every two contiguous amino acids as a combination. Therefore, there are L−1 combinations in a protein sequence. If the protein sequence information is known, we can calculate the frequency of these combinations and represent them by a 400-dimensional vector $E_{3}^{*}$. We can calculate the frequency value as the following formula:

(6) $$f_{mn}=\frac{N_{mn}}{L-1}\;,\;\;\;1\leq m\leq 20,\;1\leq n\leq 20$$

where $N_{mn}$ represents the number of combinations that consist of the mth and nth types of amino acids and appear in the protein sequence.

### 3.3. Principal Component Analysis

PCA is an effective data analysis technique which can reduce the dimension of the raw data, eliminate some noise for promoting data processing speed, and save time. It has been widely used to process data in bioinformatics and related fields [19,47]. It can retain the main information of variable interactions when the high-dimensional sample data set is transformed into a low-dimensional space.

In this work, we obtained the fused 600-dimensional numerical vector $E=\left(E_{1}^{*};E_{2}^{*};E_{3}^{*}\right)$ by combining three numerical vectors based on the physicochemical graph energy, contact graph energy, and dipeptide composition. The multi-information fusion fused different feature information of interacting protein sequences, which may bring more noise information. Thus, on the fused feature vector, the PCA method was applied to eliminate the influence of noise and integrate useful information. Considering that some important information may be ignored if the dimension is too small, the dimension of fused feature vector E was reduced from 600 to 80 through many experiments to obtain the new feature vector and improve the prediction accuracy. After using the PCA method, the most discriminative new feature set was obtained and used as input information to train the classifier for PPI prediction tasks.

### 3.4. Weighted Sparse Representation Based Classification

In this paper, the WSRC classifier was used as the classifier for predicting PPIs, which was proposed by Lu et al. [48] in 2012. It is based on the sparse representation-based classification (SRC), uses the Gauss kernel function to measure the similarity between samples, and overcomes the shortcomings of sparse coding [20].

Considering sample data set $X\in R^{m\times n}$, it consists of n samples, and each of the samples is composed of an m-dimensional feature vector. Set L denotes the number of all classes in the sample data set. The samples belonging to the lth class can be represented by a sub-matrix $X_{l}=\left[s_{l1},s_{l2},\cdots ,s_{ln_{l}}\right]$, where $s_{li}$ means the label of the ith sample belonging to the lth class and $n_{l}$ refers to the sample size of the lth class. Therefore, the sample matrix can be represented as $X=\left[X_{1},X_{2},\cdots ,X_{L}\right]$.

Assuming test sample $y\in R^{m}$ is a sample of the lth class, y can be expressed as:

(7) $$y=b_{l,1}s_{l1}+b_{l,2}s_{l2}+\cdots +b_{l,n_{l}}s_{ln_{l}}$$

This equation can be further expressed as:

(8) y=Xβ

where $\beta =\left[0,\cdots ,0,\beta_{l1},\beta_{l2},\cdots ,\beta_{ln_{l}},0,\cdots ,0\right]$. As the number of samples grows larger, α becomes sparser, since the non-zero entries in β are only related to the lth class. The key to the principle of WSRC is to calculate the vector β, which not only needs to satisfy Equation (8), but also minimize the L1-norm of β. This can be expressed as follows:

(9) $$\hat{\beta_{1}}=\arg min{\left\|W\beta \right\|}_{1},\;\mathrm{subject}\;\mathrm{to}\;{\left\|y-X\beta \right\|}_{2}<\varepsilon$$

where ε>0 is a threshold, and *W* is a block-diagonal matrix:

(10) $$diag\left(W\right)=\left[d_{G}\left(y,x_{1}^{1}\right),\cdots ,d_{G}\left(y,x_{n_{L}}^{L}\right)\right]$$

where $x_{i}^{j}$ denotes the ith sample of the jth class and $d_{G}\left(\cdot ,\cdot \right)$ represents the Gaussian distance function:

(11) $$d_{G}\left(y,x_{i}^{j}\right)=e^{-{\left\|y-x_{i}^{j}\right\|}^{2}/2\sigma^{2}}$$

where σ is the Gaussian kernel width, and $i=1,\ldots ,n_{L},j=1,\ldots ,L$. Then, the type of test sample y will be determined by the sparse representation classifier, and the formula can be described as follows:

(12) $$\underset{c}{\min}r_{c}\left(y\right)={\left\|y-X{\hat{\beta }}_{1}^{c}\right\|}_{2}$$

where c=1,…,L. In this paper, the WSRC classifier was applied for sample classification.

## 4. Conclusions

In this paper, we introduce graph energy to encode protein sequences and present a novel prediction method called PPI-GE for predicting PPIs using amino acid sequences alone. In the process of feature extraction, we designed two new feature extraction methods: physicochemical graph energy based on the ionization equilibrium constant and isoelectric point of amino acids and contact graph energy based on the contact information of amino acids. In addition, the dipeptide composition method was used to extract and supplement effective order information. These feature extraction methods can comprehensively consider the physical and chemical properties as well as the contact and location information of amino acids. The WSRC classifier was used as the classifier of the prediction model. The proposed method was tested on three benchmark data sets (human, *H. pylori*, and yeast data sets), two important PPI networks (Wnt-related pathway and Ras-Raf-Mek-Erk-Elk-Srf pathway), and five independent data sets (*H. pylori*, *H. sapien*, *C. elegans*, *M. musculus*, and *D. mela* data sets); good prediction results were obtained. The experimental results indicate that our proposed method is robust and superior compared to previous methods.

## Figures and Tables

**Table 1:** five-fold cross-validation results on the human data set.

Testing Set	ACC (%)	SEN (%)	MCC (%)	Pre (%)	AUC (%)
1	99.33	98.76	98.66	99.87	99.99
2	99.63	99.34	99.26	99.87	100
3	99.57	99.22	99.14	99.87	100
4	99.39	99.24	98.77	99.49	99.98
5	99.51	99.49	99.02	99.49	99.99
Average	99.49	99.21	98.97	99.72	99.99

**Table 2:** five-fold cross-validation results on the *H. pylori* data set.

Testing Set	ACC (%)	SEN (%)	MCC (%)	Pre (%)	AUC (%)
1	95.55	98.21	91.24	92.88	99.27
2	97.94	98.26	95.89	97.59	99.34
3	98.11	99.65	96.27	96.64	98.94
4	97.94	97.69	95.88	98.34	99.41
5	96.23	97.32	92.46	95.41	99.01
Average	97.15	98.23	94.35	96.17	99.19

**Table 3:** five-fold cross-validation results on the yeast data set.

Testing Set	ACC (%)	SEN (%)	MCC (%)	Pre (%)	AUC (%)
1	99.60	99.18	99.20	100	100
2	99.46	98.95	98.93	100	100
3	99.55	99.20	99.11	99.91	100
4	99.51	99.00	99.02	100	100
5	99.69	99.38	99.38	100	100
Average	99.56	99.14	99.13	99.98	100

Note: ACC: accuracy; SEN: sensitivity; MCC: Matthews correlation coefficient; Pre: precision; AUC: area under the curve.

**Table 4:** Comparison of different methods on the Wnt-related network using yeast data set as the training data set.

Wnt-Related Network	Proportion	Accuracy (%)
Proposed method	92/96	95.83
Ding’s work [30]	89/96	92.71
Shen’s work [35]	73/96	76.04
Zhou’s work [36]	87/96	90.63
Chen’s work [29]	89/96	92.71

**Table 5:** Comparison of the accuracy (%) between different methods on the independent data sets using yeast data set as the training data set.

Data Set	Testing Pairs	Proposed Method	Huang’s Work [34]	Du’s Work [37]	Ding’s Work [30]
*H. pylori*	1420	93.80	85.77	93.66	92.03
*H. sapien*	1412	99.93	88.81	93.77	94.58
*C. elegans*	4013	86.24	72.79	94.84	90.28
*M. musculus*	313	94.57	83.39	91.37	92.25
*D. mela*	21975	99.87	89.35	N/A	N/A

Note: N/A means not available.

**Table 6:** The details of three benchmark data sets.

Datasets	Protein Pairs	Interaction Pairs	Non-Interaction Pairs	References
human	8161	3899	4262	[20]
*H. pylori*	2916	1458	1458	[38]
yeast	11,188	5594	5594	[18]
